# Short-Term Supplementation of Sauerkraut Induces Favorable Changes in the Gut Microbiota of Active Athletes: A Proof-of-Concept Study

**DOI:** 10.3390/nu16244421

**Published:** 2024-12-23

**Authors:** Andrija Karačić, Jadran Zonjić, Ena Stefanov, Katja Radolović, Antonio Starčević, Ira Renko, Željko Krznarić, Matija Ivančić, Zvonimir Šatalić, Ana-Marija Liberati Pršo

**Affiliations:** 1 Faculty of Food Technology and Biotechnology, University of Zagreb, Pierottijeva 6, 10000 Zagreb, Croatia; andrija@ccm.hr (A.K.); jadran.zonjic@gmail.com (J.Z.); enastefanov44@gmail.com (E.S.); antonio.starcevic@gmail.com (A.S.); ira.renko@gmail.com (I.R.); zvonimir.satalic@pbf.unizg.hr (Z.Š.); 2 The Gut Microbiome Center (CCM), Jablanska 82, 10000 Zagreb, Croatia; 3 Department of Internal Medicine, University Hospital “Sveti Duh”, Sveti Duh 64, 10000 Zagreb, Croatia; matijai2307@gmail.com; 4 Department of Internal Medicine, Faculty of Medicine, University of Zagreb, 10000 Zagreb, Croatia; zeljko.krznaric60@gmail.com

**Keywords:** sports nutrition, sauerkraut, microbiome, fermented food, whole food, athletes

## Abstract

Background: Since the gut microbiota is important for athlete health and performance, its optimization is increasingly gaining attention in sports nutrition, for example, with whole fermented foods. Sauerkraut is a traditional fermented food rich in pro-, pre-, and postbiotics, which has not yet been investigated in the field of sports nutrition. Methods: To determine whether sauerkraut could be used for gut microbiota optimization in sports nutrition, a proof-of-concept study was conducted. The microbiota composition of organic pasteurized sauerkraut was analyzed, and then healthy active athletes were provided with the same sauerkraut for 10 days as an intervention. The effects of sauerkraut on the athlete’s gut microbiota, laboratory parameters, and bowel function were assessed. Results: Significant changes in the gut microbiota composition were seen on taxonomic and functional levels, independent of baseline microbiota composition, even after short-term supplementation. Most notably, there was an increase in several health-promoting genera of the family *Lachnospiraceae*, as well as significant alterations in metabolic pathways regarding cell wall synthesis and the metabolism of nucleotide bases. An increase in the proportion of lymphocytes and a decrease in B12 vitamin levels was observed, as well as a risk of indigestion in certain athletes, which significantly resolved after seven days of supplementation in all athletes. It is unclear whether the observed effects are attributable to the sauerkraut’s own microbiome or its pre- and postbiotics since it is a whole food. Conclusions: Our study has demonstrated that the concept of whole fermented foods, such as sauerkraut, could potentially be feasible and effective in sports nutrition for gut microbiota optimization.

## 1. Introduction

The gut microbiota refers to the complex microbial ecosystem in our digestive tract [1], which has a major role in host physiology [2]: from metabolism and immunity to the brain and nervous system [3]. Due to extreme interindividual variability, with only 10% of the gut microbiota being shared between individuals, there is no standard gut microbiota composition [4]. But it is widely agreed that a diverse, balanced, and highly functional gut microbiota, which is abundant in health-promoting bacteria and lacks potentially pathogenic and pro-inflammatory bacteria, is ideal for optimal health [4].

The intentional optimization of gut microbiota composition and functionality with gut-microbiota-targeted therapeutics, such as probiotics, prebiotics, and postbiotics, is traditionally investigated in the context of the prevention and treatment of various infectious, metabolic, and autoimmune diseases, especially due to the immunomodulatory effects on systemic inflammation [5]. Probiotics are live microorganisms that exert beneficial health effects upon consumption [6], prebiotics are substrates that are utilized selectively by the gut microbiome for growth [7], and postbiotics are inanimate microorganisms and/or their components that confer a health benefit on the host [8].

Recently, an increasing interest in gut microbiota optimization in the field of sports nutrition has been seen [9]. No wonder, since the gut microbiota is essential for athlete health, performance, and especially, recovery [10,11]. Its metabolites, such as short-chain fatty acids (e.g., butyrate), lactic acid, and neuroactive metabolites (GABA, serotonin), directly and indirectly via immune, hormonal, or nervous signaling, improve the functioning of the gut–muscle, gut–joint, gut–liver, and also gut–brain axes, which are all crucial for athletic performance, as well as recovery [12,13]. A well-known example in this context is *Veillonela atypica*, discovered in active marathon runners [14], which converts exercise-derived lactate into propionate, which can serve as a substrate for gluconeogenesis in the liver, a phenomenon also found in other genera [15]. On the other hand, regular physical activity is beneficial for gut microbiota, hence rendering the gut microbiota of athletes more diverse and functional than those of sedentary individuals [16,17,18].

To the best of our knowledge, research on the field of gut microbiota optimization in athletes was focused mainly on probiotics, with prebiotics being mostly neglected and synbiotics such as whole fermented foods even more. Current evidence suggests that probiotics are effective in athletes solely regarding recovery and prevention of respiratory and gastrointestinal infections.

Sauerkraut is rich in pro- and prebiotics [19]. It is probably the most popular fermented whole food preparation in Europe [20]. Sauerkraut is a fermented vegetable product that is derived from the malolactic fermentation of raw fresh white cabbage (*Brassica oleracea* L. var. *capitata*) in a salt brine with 2–3% (*w*/*w*) sodium chloride. Since the fermentation process involves several live beneficial lactic acid bacteria (LAB) naturally present in fresh cabbage or in the food processing environment, sauerkraut is regarded as a probiotic-rich food. Some of them are *Weissella* spp., *Leuconostoc mesenteroides*, *Levilactobacillus brevis*, *Lactiplantibacillus plantarum*, and *Pediococcus pentosaceus* [21]. The literature reports that these probiotic bacteria are resistant to bile salts and low gastric pH (one even to β-hemolysis) and demonstrate antimicrobial activity [22]. During food fermentation, controlled bacterial metabolism (activity and growth) on the medium converts fermentable substrates, mainly carbohydrates and proteins, into biologically active metabolites, including short-chain fatty acids (SCFAs) and biogenic amines. Besides that, sauerkraut is rich in fiber by nature, which can act as a prebiotic. Therefore, sauerkraut can be regarded as a synbiotic whole food whose beneficial effect is due to all of its compounds: pro-, pre-, and postbiotics.

The health benefits of sauerkraut consumption have been studied in a limited body of research, mostly on in vitro models [23]. Human clinical trials are still scarce. Nevertheless, the consumption of fermented foods bearing similar properties to sauerkraut, such as kimchi, has been correlated in sports nutrition with improvements regarding immunity and metabolism, specifically an increase in anti-inflammatory pathways and a decrease in fasting blood glucose levels in diabetes [24,25,26]. The underlying cause of the observed effects can probably be attributed to the ingestion of certain probiotic bacteria and bioactive compounds, such as the fiber found in the starting food material and the fermentation-related metabolites [27]. Especially short-chain fatty acids (SCFA), such as butyrate and biogenic amines, and natural polyamines (putrescine, spermine, spermidine), have been shown to induce a plethora of beneficiary effects in the gastrointestinal tract (intestinal motility, barrier function, energy source) and immune system (anti-oxidative, anti-inflammatory effects) [28].

Since no data on the potential of sauerkraut in sports nutrition specifically were found, especially in the context of gut microbiota optimization, a gap in knowledge was detected. A proof-of-concept study was conceived to explore the potential of sauerkraut for gut microbiota optimization in active athletes. The hypotheses are that sauerkraut is, on one hand, a synbiotic, rich in beneficial bacteria and prebiotics, and on the other hand, effective in inducing significant favorable changes in the gut microbiota composition and functionality, independent of baseline gut microbiota status and even when supplemented for a short period of time. Besides the gut microbiota, the aim was to assess the effects of short-term supplementation on bowel function and routine laboratory parameters to gain insight into the risks and feasibility of sauerkraut supplementation in sports nutrition. The purpose of this preliminary study was to obtain data that would guide high-quality future research on this topic.

## 2. Materials and Methods

### 2.1. Study Design

The present study was conceived as a preliminary proof-of-concept study. A cohort of professional athletes was followed over a short course of sauerkraut supplementation, where its effects were monitored by gut microbiota and laboratory analyses. For the intervention, a duration of 10 days was picked. The reasoning for such a short intervention time was twofold. On the one hand, it was to prove that sauerkraut is indeed an effective synbiotic which does not require extensive time for action, similar to other studies [29,30]. On the other hand, due to the highly variable schedule of professional athletes long-term monitoring (e.g., a month) of potential confounding factors, such as diet and training, was impossible. to.

Participants were recruited in collaboration with the Croatian Olympic Committee. The goal was to recruit professional athletes of different athletic disciplines to yield a more general overview. The inclusion criteria were (1) adult age, (2) male gender, (3) either status of a professional athlete by standards of the Olympic Committee or professional engagement in non-Olympic sports, and (4) good general physical health (as assessed by the annual health check-up by the Olympic Committee Medical Commission or primary medical care). Exclusion criteria were: (1) administration of antibiotics at least six months prior or during the intervention, (2) supplementation with probiotics at least six months prior or during the intervention, (3) chronic medical therapy, (4) known intolerance to sauerkraut or cabbage intake, and (5) previous intraabdominal surgery. Volunteers fulfilling the inclusion criteria visited the Gut Microbiome Center for an initial screening, where they received a detailed verbal explanation and written instructions from the team of researchers. A written informed consent form was obtained after all methods, risks, and benefits were thoroughly explained. The participants were handed the kits for the stool sampling and the sauerkraut. Body composition was assessed prior to intervention using a TANITA Body Composition Analyzer MC-780 (Tanita Corporation, Tokyo, Japan).

The participants were asked to record all the food, beverages, and dietary supplements they consumed using a 7-day food record before the start of the intervention and a 10-day food record during the intervention, as well as the Athlete Diet Index Questionnaire (ADI) [31], to evaluate their nutritional and lifestyle behavior (fiber intake, frequency, and intensity of physical activity) and energy intake. The day prior to the start of the intervention, the participants provided a stool sample following the instructions at their home. The participants were instructed not to alter their lifestyle and dietary behavior throughout the intervention and to report it in the online form.

The intervention consisted of the daily supplementation of 250 g of sauerkraut, independent of body mass, over the course of ten days. The participants were allowed to have variations in the daily amount and the time of intake during the day depending on the situation in the given days due to self-rationing and reported the quantity of ingested sauerkraut and time of intake in the online form. The participants consumed the sauerkraut throughout the day, either alone or in combination with other food in a meal (salad, side dish). The participants were asked to report whether they had experienced any adverse effects consequently. The day after the end of the intervention, the participants provided the second stool sample, which was sent off to the laboratory, and once again completed the ADI. The present study took place during the off-season for all athletes.

### 2.2. Participants

A minimum of ten active athletes was defined as the minimal study population to yield significant results. All participants completed detailed food records regarding dietary intake, including lifestyle (sleep, physical activity) prior to and during the intervention. The participants noted daily the amount and time of sauerkraut supplementation and the incidence of any adverse effects which could potentially be associated with the sauerkraut intake.

All procedures relative to this study were in accordance with the Helsinki Declaration and approved by the Ethics Committee of the School of Medicine, University in Zagreb, for the protection of human subjects (ref. number 380-59-10106-23-111/36) on the date 27 March 2023. This study was registered on ClinicalTrials.gov under the number NCT06087146.

### 2.3. Supplementation Protocol

The sauerkraut used in this study was produced by Eko Imanje Zrno Ltd., Vrbovec, Croatia. The sauerkraut was locally sourced, grown under organic conditions, and prepared by fermentation using a salt brine without the use of preservatives and pasteurized. The sauerkraut was packaged in glass jars in portions of 500 g. Every participant received five jars of sauerkraut. Before the commencement of the study, sauerkraut was tested for its snutritional and microbiological properties in accredited laboratories of the School of Biotechnology and Food Technology of the University of Zagreb. The nutritional information is enclosed in Table 1. In cultures, the count of lactic-acid-forming bacteria was 4.82·10^3^ (±2.31) CFU/mL. In cultures, no harmful microorganisms were detected, including *Staphylococcus aureus*, *Enterobacteriaceae*, sulfite-reducing *Clostridia*, and fungi (<10 CFU/g). The sauerkraut was tested for its microbiome composition by 16s rRNA amplicon sequencing using Illumina NGS (Biomes NGS Ltd., Wildau, Germany). Five samples were taken for analysis according to the following sampling protocol: two samples of sauerkraut brine of equal quantity (10 mL), one 5 mL sample of sauerkraut brine, and two samples containing 6 g and 3 g, respectively, of sauerkraut itself in addition to 5 mL of brine.

### 2.4. Standardization of Physical Activity, Sleep, and Diet

Physical activity and diet were evaluated using a specially designated online form in Excel (Microsoft, Palo Alto, CA, USA). Participants entered the start and end, as well as the type of each training session, seven days before and the ten days of the intervention. Dietary intake was monitored during the same period in the two phases, and participants noted the time, quantity, and ingredients of every ingested food item following the instructions by the researchers. If the participants were not able to weigh the quantity of a specific food item, they utilized the Capnutra food atlas to indicate food quantity. The data were then analyzed by the researchers (dietitians), and food intake was classified regarding macronutrient and energy intake as average values before and during intervention using USDA food composition databases. The Athlete Diet Index (ADI) served as a measure of convergent validity to assess potential changes in diet before and during intervention. The ADI is a valid and reliable diet quality assessment tool in the form of an online questionnaire. It was developed purposely for active athletes and evaluates regular dietary intake, especially regarding nutrients relevant to athletes, compared with sports nutrition recommendations by generating an overall score of the athlete’s diet [31].

### 2.5. 16S rRNA NGS of the Gut Microbiota

Stool samples were taken the day before and one day after the end of supplementation (Day 0 and Day 11) using the intest.pro at-home testing kit developed by Biomes NGS Ltd. The day before and the day after the intervention, the participants took stool samples using a cotton swab of feces off the toilet paper after swiping at their home following the instructions, and the swabs were conserved in up to 1000 µL of a proprietary DNA-stabilizing buffer containing Tris, developed by Biomes NGS Ltd. The samples were transported by logistical services the next workday over the course of a couple of days to the laboratory (Biomes NGS, Wildau, Germany). Upon arrival, the stool samples were stored at −20° C until sequencing. For the lysis process, the samples were defrosted and centrifuged at 400× *g* for 15 min. Afterward, 650 µL of warmed-up lysis buffer was added to each sample and then vortexed for 20 min. Afterward, the nucleic acids were extracted on a liquid handling system (Hamilton StarLine (Hamilton Company, Reno, NV, USA) and Tecan EVO (Tecan Group Ltd., Männedorf, Switzerland)) by using a vacuum chamber, as well as a high-pressure chamber. The extracted gDNA was stored at −20 °C until use. The library preparation followed the manual “16S Metagenomic Sequencing Library Preparation—Preparing 16S Ribosomal RNA Gene Amplicons for the Illumina MiSeq System”. For the normalization of all samples, a fluorescent dye and the Biotek Synergy HTX plate (Biotek Instruments, Winooski, VT, USA) reader were used to measure DNA concentrations and to calculate the necessary dilution volume per sample. All the steps described are nearly fully automated by using a liquid handling system (Hamilton StarLine (Hamilton Company, Reno, NV, USA)), allowing for parallel sample processing. The library denaturing and MiSeq sample loading were carried out manually following the Illumina protocol for MiSeq Reagent Kit v3 (600-cycle) (Illumina, San Diego, CA, USA). Demultiplexing was performed directly on the platform, using MiSeq Reporter Analysis software v2.6 (Illumina, San Diego, CA, USA), right after sequencing, and the resulting FastQ files were generated for subsequent data analysis.

For the processing and analysis of sequence data, the Paired-End-Reads from MiSeq (2 × 300 cycles) were merged to reconstruct overlapping sequences with 430–460 base-length. Chimera and borderline reads were filtered out with the usearch uchime2_ref tool. SILVA 138.1 is used as a Database for usearch uchime2_ref. For taxonomic alignment, Amplicon sequence variants (ASVs) were determined using BLASTn (Nucleotide-Nucleotide BLAST 2.10.1+) against SILVA 138.1 [32]. BLAST was utilized due to its high accuracy in sequence alignment, which ensures reliable ASV identification and homologous region detection, its flexible customization options, and the fact that it is widely used, well documented, and seamlessly integrated with extensive databases like NCBI. Alignment identity must meet a threshold of at least Phylum: 75.0%, Class: 78.5%, Order: 82.4%, Family: 86.5%, Genus: 94.5%, and Species: 97.0%. RefSeqs/Counts tables were created for all samples using the Python package Pandas 1.3.4. The taxonomic composition of microbial communities is inferred from ASV (Amplicon sequence variants) counts at the phylum, genus, and species level. For the prediction of functional profiles, the Picrust2 tool was utilized [33]. The study sequences of the alignment step were placed into a reference tree to determine/predict the copy numbers and the NSTI-index (Nearest-sequenced taxon index). All study sequences with an NSTI-index higher than 2 were excluded.

This same method was utilized to analyze the sauerkraut and its constituents (cabbage, brine) for its microbiome composition present in the product delivered to the participants.

### 2.6. Laboratory Analysis

The day before and after the end of the intervention, a laboratory analysis was performed to assess potential changes in the participant’s physiology associated with the sauerkraut supplementation. The laboratory measures taken were as follows:

1. Blood count: erythrocytes, leukocytes, neutrophils, lymphocytes;
2. Metabolism: serum low-dense lipoprotein cholesterol levels (LDL), uric acid levels;
3. Hormone levels: thyroid (TSH, FT3), testosterone, blood glucose (insulin, HOMA-IR), cortisol;
4. Vitamins: vitamin D, B12, folic acid.

The laboratory analysis was performed at a tertiary health care facility using EDTA or citrate blood depending on the respective measure on a standard laboratory setup using chemiluminescence immunoassay (CLIA), electrochemiluminescence immunoassay (ECLIA), and enzymatic tests depending on the respective laboratory parameter. The assays were performed in accordance with the manufacturer’s protocols, with internal and external quality controls to ensure accuracy, and results were interpreted based on established reference ranges.

### 2.7. Statistical Analysis

For the analysis, all online forms were coded, and the data were imported into SPSS (IBM Corp. Released 2020. IBM SPSS Statistics for Macintosh, Version 27.0. Armonk, NY, USA: IBM Corp). Descriptive and inferential statistical analyses were conducted.

Data on dietary intake and lifestyle were quantitative variables. After they were tested for the distribution type using the Kolmogorov–Smirnov test, the results of the descriptive analyses were presented as median and interquartile range. Differences in the distributions of the quantitative variables were analyzed with Mann–Whitney U tests.

Statistical analysis of the results of the gut microbiota analyses was performed. For alpha-diversity calculations, ASV counts were rarefied to 10.000 counts per sample. Shannon index was chosen as the alpha-diversity metric and calculated using the diversity function provided by Qiime2 [34]. In addition, the relative abundances of the *Lactobacillus* Group and of the Phylum *Proteobacteria* were evaluated in relation to sauerkraut intervention. A Repeated Measures Correlation was performed for hypothesis testing using the R function rmcorr [35]. Calculated *p*-values were adjusted for multiple testing using Benjamini and Hochberg correction, and significance was assumed at a *p*-value < 0.05 (non-FDR) [36].

To account for the compositional properties of relative ASV abundances, a centered-log-ratio (clr) transformation was performed in preparation for further analyses. As the logarithm of zero is undefined, all values were summed with a small pseudocount, which amounts to the smallest non-zero value divided by 10. To visualize compositional differences between treatment groups, a principal component analysis was conducted using the PCA function of the R package FactoMineR 2.11 [37].

To identify differentially abundant genera and pathways in regard to sauerkraut intervention, a Repeated Measure Correlation was performed using the R package rmcorr 0.7.0 [26]. Analysis was carried out on Genus and Pathway level using a filtered table with a cutoff at 0.1% average relative abundance across all samples. To account for compositionality, centered-log ratio transformation was applied using a pseudocount of the smallest non-zero value divided by 10. Calculated *p*-values were adjusted for multiple testing using Benjamini and Hochberg correction, and significance is assumed at a *p*-value < 0.05 (non-FDR).

The R package ggplot2 3.5.1 was used for visualization [38].

For the statistical analysis of the five sauerkraut samples, PERMANOVA analysis (permutational multivariate variance analysis) and Kolmogorov–Smirnov test were utilized in Python using Pandas and SciKit libraries to test whether the samples containing brine were similar to each other and whether they significantly differed from the samples containing the sauerkraut itself regarding the distribution of relative abundances of bacteria.

In order to calculate a 95% confidence interval for the probability of Bristol stool type to be 3 or 4 during the period of ten days, a binomial test was performed in R programming language. In the same programming language, the probability of Bristol stool type to be 3 or 4 for each day was calculated using the chi-squared test. As statistically significant values were taken, ones that *p*-value was lower than 0.5. Hazard ratio was obtained by putting the number of participants that had Bristol stool type 3 or 4 into ratio compared with the number of all the participants for each day.

## 3. Results

### 3.1. Sauerkraut Microbiota

The 16s rRNA gene amplicon sequencing of the five sauerkraut samples revealed 1416 taxa and a total of 515.608 aligned reads, at average 103,121.6 ± 53,380.8. The ten most abundant bacterial genera were, in order of abundance, *Bacteroides*, *Blautia*, *Faecalibacterium*, *Prevotella*, *Ruminococcus*, *Roseburia*, *Agathobacter*, *Fusicatenibacter*, *Lachnospiracae (unspecific)*, and *Subdoligranum*. The added relative abundances of these ten genera accounted for around 50% in four of the five samples (all brine samples and the sample with 50% sauerkraut). All the aforementioned predominant genera of the sauerkraut’s microbiota are obligate aerobes and are highly metabolically active. Utilizing carbohydrate-active enzymes (CAZymes), such as glycoside hydrolases, and fermentative enzymes, such as butyrate and acetate kinase, these genera are known to produce SCFA, such as butyrate [39]. The relative abundances of taxa commonly involved in food fermentation were extremely low: from 0.03% to 0.09% for the *Lactobacilliales* family, and from 0.001% to 0.02% for genus *Leuconostoc*. The genus *Pediococcus* was not even detected in any of the samples.

### 3.2. The Intervention

The CONSORT extension for pilot and feasibility studies, as well as the SPIRIT Standard Protocol Items: Recommendations for Interventional Studies guidelines, was utilized in designing the study protocol and results reporting.

This study was conducted over the course of six months in a collaboration between several organizations: The Gut Microbiome Center in Zagreb, Croatia, the Faculty of Food and Biotechnology, University of Zagreb, and the Laboratory of Biomes NGS Ltd., Wildau, Germany.

The characteristics of the participants are shown in Table 2a, including gender, age, type of sport, and years of sport participation. Regarding the classification of participants based on their level of physical activity and sport performance, participants were classified using the 6-tiered Participant Classification Framework. Although the plan was to conduct the study on only male athletes, one female participant was included since the principal aim of this study was to perform the analysis on professional athletes, and no additional interested male athletes were found.

The physical characteristics (height and body mass) and body composition variables (fat-free mass, skeletal muscle mass, percentage of body fat, and fat mass) are shown in Table 2b. The data from participant 3 could not be retrieved due to technical issues.

Physical activity and sleep were monitored before and during the intervention. Data on frequency and average duration of physical activity and sleep quantity are disclosed in Table 2c. No statistically significant differences before and during the intervention were registered.

All aspects of dietary intake were monitored before and during the intervention. Data on average daily dietary intake are disclosed in Table 2d. Besides the significant increase in daily fiber per 1000 kcal intake during the intervention, no other statistically significant differences regarding food intake before or during the intervention were registered. These observed changes could be attributed to the sauerkraut intake, since it is itself rich in fiber but low in calories. Due to certain specific dietary habits recorded by the participants (e.g., fast food and prepackaged foods for which nutritional information is difficult to obtain) and disparities in nutritional information present in food composition tables and food declarations, the aforementioned dietary intake results are only estimates and should be taken with caution.

Not all participants completed all subscales of the ADI due to bad compliance. The data for those who completed it show that there were no significant differences before and during the intervention (Table 2e). The detected low fiber intake and a relatively low carbohydrate intake by the participants are in accordance with their qualitative ADI results, which hint at an insufficient grain intake among all participants.

The food records and the ADI questionnaire showed independently that the participants did not significantly alter their dietary intake during the intervention.

### 3.3. Gut Microbiota

**Figure 1:**
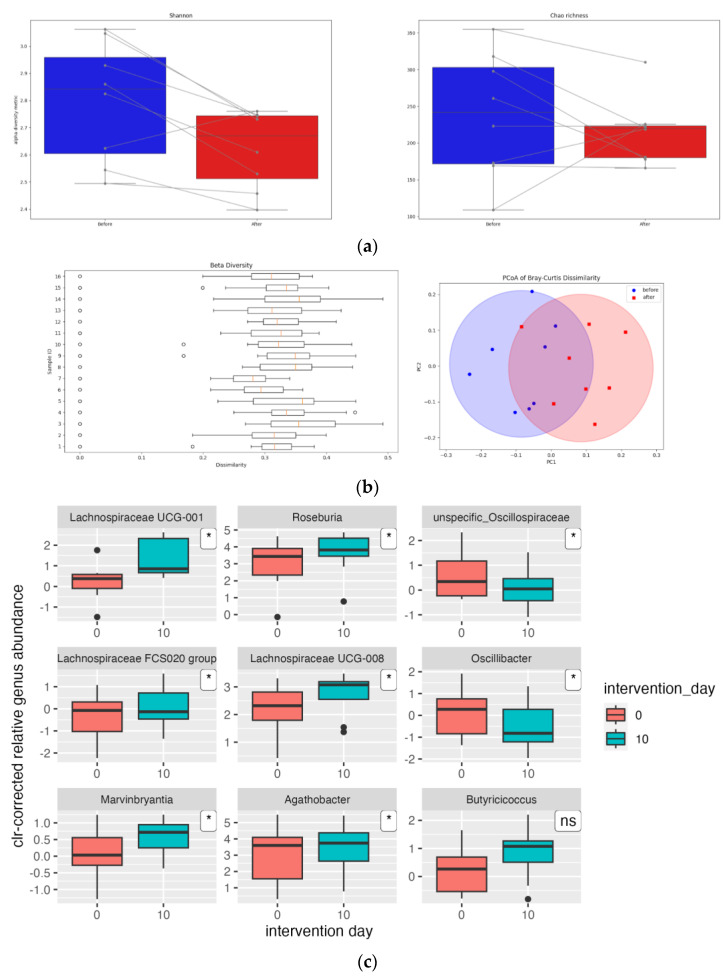
Visual representation of effects of sauerkraut supplementation on alpha and beta diversity and bacterial genera: (**a**) Changes in alpha-diversity metrics Shannon index and Chao richness after sauerkraut supplementation. (**b**) Beta diversity: distribution of Bray–Curtis dissimilarity values among samples and Principal Coordinates Analysis (PCoA). (**c**) Significant changes in centered-log-transformed (clr) relative abundances of bacterial genera after intervention (* indicating *p* < 0.05; ns non-significant).

The results of the fecal microbiota analyses were analyzed regarding alterations in relative abundances of bacterial counts grouped either in taxonomic or functional groups, therefore indicating changes in gut microbiota composition and functionality. A presentation of general results is disclosed in Figure 1.

No significant differences were found in the α-diversity of the gut microbiota of the study population due to sauerkraut intervention (Figure 1a). In order to see if the sauerkraut had an impact on gut microbiota composition, beta diversity was calculated for the taxonomy levels of genus and phyla using Bray–Curtis metrics. The level of significance was obtained using ANOSIM [40]. The results show that there was a significant difference in gut microbiota composition (*p* < 0.001) post sauerkraut supplementation. However, an R-value of 0.314 indicates that the difference is moderate (Figure 1b). Although several significant taxonomic changes were seen post-intervention, due to weak significance rates and high false positivity rates, the observed associations have to be considered with caution. At the phylum level, no significant changes were seen post intervention regarding the major phyla such as Firmicutes and Bacteroidetes, only a significant decrease in the relative abundance of phylum Verrucomicrobia (*p* = 0.007, q = 0.074). At the family level, the most notable changes were decreases in the relative abundances of Akkermansiaceae (*p* = 0.049) and Oscillospiraceae (*p* = 0.065) and increases in Lachnospiraceae (*p* = 0.058), Butyricicoccaceae (*p* = 0.094), and Clostridiaceae (*p* = 0.096). It is important to note that the q-values (false-discovery-rate-corrected *p*-values) for the aforementioned effects were 0.769. On the level of genera, however, after the intervention, significant changes in bacterial counts on taxonomic levels were seen in eight genera (Figure 1c). Increases in genera belonged to the *Lachnospiraceae* family: *Lachnospiraceae UCG-008*, *Lachnospiraceae UUCG-001*, *Roseburia*, *Lachnospiraceae FCS020 Group*, *Marvinbryantia,* as well as *Agathobacter* (formerly known as *Eubacterium rectale*), and decreases in genera belonged to the *Oscillospiracaeae* family: unspecific *Oscillospiraceae* and *Oscillibacter*.

### 3.4. Gut Microbiota Functionality

A total of 190 metabolic pathways were analyzed among the identified bacterial groups. Significant changes in bacterial counts in 35 metabolic pathways were found (18.4%). These changes exclusively affected pathways that concern cell wall synthesis and the metabolism of nucleotide bases (purine, pyrimidine, etc.) and were negatively correlated to the sauerkraut intake. Bacteria actively involved in these pathways were significantly affected by the intervention, with low rates of false positive observed changes. The pathways significantly affected by the sauerkraut intervention are disclosed in Table 3a.

To better understand the functional dynamics of microbial communities, we grouped the functional metabolic pathways predicted with PiCRUST2 into functional modules using MetaCYC based on their class, ontology, or interpretation. Several significant changes were observed. After grouping, we found that the metabolic pathways involved in polysaccharide and sugar degradation, as well as protein fermentation, were enhanced after intervention. At the same time, metabolic pathways involved in butyrate metabolism and inflammation were decreased. However, after adjustment for multiple testing, the false discovery rate (FDR) showed no significant differences in predicted functionality between pre- and post-intervention (Table 3b).

### 3.5. Laboratory Analyses

A spectrum of laboratory parameters from blood samples were assessed over the course and after the intervention. Data on the average values before and the day after are disclosed in Table 4. Besides an increase in serum lymphocyte counts and a decrease in serum B12 levels, no significant short-term changes in laboratory parameters were seen after the intervention.

### 3.6. Bowel Movement and Adverse Effects

Participants reported bowel movements and any potential adverse effects through their study diary. Results are disclosed below in Table 5. Although at the beginning of the intervention, different types of Bristol stool types (BST) were reported, from Day 8 to Day 10, all participants but one reported having BST 3 or 4, which are considered physiological forms and consistencies of stool. Statistical analysis showed that the probability for BST 3 and 4 significantly increased after a week of sauerkraut consumption. A minority of participants reported bloating, diarrhea, and pain during the intervention, and no episodes of constipation were reported. The largest number of adverse effects was reported around Days 5 to 6.

## 4. Discussion

The current study confirmed the two initial hypotheses. Organic, pasteurized sauerkraut is potentially an effective synbiotic rich in health-promoting anaerobes. When around 250 g of it is supplemented over the course of only ten days, it induces several significant changes in the composition and functionality of the gut microbiota in active athletes, as well as significant changes in the proportion of lymphocytes and levels of vitamin B12. But its supplementation is associated with a risk of indigestion, which resolves after a week of administration. Therefore, sauerkraut could indeed be utilized in sports nutrition for gut microbiota optimization in all athletes, confirming the concept of sauerkraut as a potential synbiotic food product for athlete health and performance.

The 16S rRNA sequencing of the sauerkraut microbiota surprisingly showed minimal relative abundances of bacteria commonly involved in food fermentation, such as *Lactobacilliales*, *Leuconostoc,* and *Pediococcus*, contrary to other studies [41,42]. Even when analyzed by conventional microbiological cultures, the LAB counts were smaller than expected. This implicates setbacks related to the performed sampling and sequencing methods, especially since the composition resembles the human gut microbiota. Therefore, should the data on the sauerkraut microbiota composition should be interpreted with great caution.

Contrary to similar studies, where fermented foods were found to increase the alpha-diversity of the gut microbiota [43,44], sauerkraut supplementation did not result in an increase in alpha-diversity. Two measures of alpha-diversity, the Simpson index and Chao richness, showed a non-significant reduction in their median values. Due to the small sample size and the short duration of the intervention, the underlying mechanism can only be speculated upon.

Regarding taxonomic changes, sauerkraut supplementation seems to invoke a specific shift in the gut microbiota composition: an increase in families producing short-chain fatty acids from fiber fermentation, such as *Lachnospiraceae*, *Butyricicoccaceae*, and *Clostridiaceae*, and a decrease in the *Akkermansiaceae* (belonging to the significantly reduced phylum Verrucomicrobia) and *Oscillospiraceae* families. This is supported by statistically significant results regarding beta diversity (Figure 1b). It is important to note that these changes were independent of baseline gut microbiota composition, since these changes were seen in all participants and in the same direction.

Complementary studies showed that the *Blautia* and *Roseburia* species, often associated with a healthy state of the microbiota, are some of the main SCFA producers. *Blautia* and *Roseburia* are health-promoting bacteria, since they are the genera most involved in the control of gut inflammatory processes, atherosclerosis, and maturation of the immune system [45], demonstrating that the end-products of bacterial metabolism (butyrate) mediate these effects.

Besides the specific changes in composition, even more significant changes were seen in the abundance of specific pathways within the gut microbiome. When considered on their own, the abundance of individual pathways associated with the metabolism of nucleotide bases was significantly reduced, as well as those involved in cell wall synthesis. When the pathways were grouped into modules based on functionality significant but potentially falsely positive, changes were also observed regarding not only the metabolism of nutrients (carbohydrate, sugars, and protein) but also related to butyrate metabolism and inflammation.

The data suggest that the changes induced by sauerkraut in the gut microbiota are potentially favorable for the individual athlete. Although sauerkraut supplementation did not render all participants’ gut microbiota more diverse (α-diversity) and balanced (Firmicutes to Bacteroidetes ratio), it increased the abundance of health-promoting SCFA-producing bacteria and made the gut microbiota more functional by changing a large number of metabolic pathways.

A possible consequence of these positive changes in the gut microbiota could be a significant increase in the proportion of lymphocytes. Although increased lymphocyte percentages are usually associated with certain pathologies, such as viral infections and hematologic and chronic inflammatory states, the values did not reach levels typical for such conditions [46]. But it is known that certain gut bacteria and their metabolites promote the proliferation of B and T lymphocytes in gut-associated lymphoid tissue (GALT) [47]. Pro-, pre-, and synbiotics have been found to enhance the immune response and stimulate lymphocyte proliferation and activity [48]. A meta-analysis of the effect of probiotic intake on white blood cell counts in 836 athletes found significantly increased leukocyte counts after the administration of multi-strain probiotics [49]. Therefore, could the observed increase potentially be a result of the changes in the gut microbiota caused by the sauerkraut supplementation? Alternatively, could the increased lymphocyte count be a stress response to the drastically increased intake of fermented food during the intervention, since an interventional study on healthy students with 100 g of kimchi during a four-week period found no significant differences in white blood cell counts [50].

Changes in the gut microbiota post sauerkraut could also be responsible for the decrease in vitamin B12 levels. Serum vitamin B12 levels depend on dietary intake [51] and production in the gut microbiota, especially by bacteria such as *Pseudomonas denitrificans*, *Klebsiella pneumoniae*, *Lactobacillus reuteri* and *plantarum*, *Clostridium difficile* and *butyricum*, and *Fusobacterium* spp., as well as *Akkermansia muciniphila* [52]. The decrease in vitamin B12 levels could be attributed to the significant decrease in the relative abundance of the *Akkermansiaceae* family, which was the most significant decrease in all bacterial families. Another explanation could be that the increase in health-promoting obligate anaerobes, such as *Roseburia* and *Blautiai,* was at the expense of a decrease in potentially pathogenic facultative anaerobes. But both explanations are highly speculative, since several bacteria are involved in B12 metabolism, and the relative abundance of bacteria does not necessarily correlate with their metabolic activity. Higher levels of vitamin B12 have been associated with the development of colorectal cancer [53], and the root of this association could lie in the gut microbiota or in an imbalance of the gut microbiota, respectively. Therefore, the decrease in vitamin B12 probably should not be regarded as a negative consequence, but on the contrary, as another positive result of sauerkraut supplementation.

It is important to note that sauerkraut intake was associated with indigestion in several participants. This is known from the literature and clinical practice, since pro- and prebiotic administration has been associated with gastrointestinal side effects, such as bloating, gaseousness, pain, and changes in bowel movement [54]. Another possible reason could be histamine intolerance, since fermented food is rich in biogenic amines such as histamine. But since these side effects of the sauerkraut supplementation waned after a week of supplementation, one could state that sauerkraut supplementation does come with a certain risk of indigestion during the first days of administration. But with regular use, digestion and the gut microbiota apparently adapt to its compounds, and then sauerkraut becomes safe to use.

There are several limitations of this study, hindering further conclusions. The main limitation of this study was the small subject number due to the many technical and organizational challenges facing studies on active athletes. This is represented by high false positive rates in the significant results of the study, especially regarding the main findings of the study—the changes in bacterial taxa. Studies on greater samples are required to yield a clearer picture of the described effects. Secondly, since a fermented food was investigated, and not its isolated compounds, this study design may not deliver insight into which specific compound in sauerkraut was most contributing to the described effects. We cannot attribute the results to specific probiotics, nor prebiotic or postibiotic compounds in sauerkraut specifically. One can even hypothesize that in the itinerated analysis of the participant’s gut microbiota, the microbiota of the sauerkraut was detected and not an altered domicile gut microbiota. A further limitation of this study’s design is the fact that it is difficult to state whether the changes seen after the intervention were positive or negative regarding athletic performance. Another question is whether the observed changes are temporary or permanent, as well as how long they persist after cessation of sauerkraut intake. Since there is a lack of information regarding a healthy human gut microbiota or effective athlete gut microbiota, for reference, we cannot conclude whether the changes seen are actually part of a so-called gut microbiota optimization. One important limitation of this study is the fact that the investigated sauerkraut was pasteurized. The process of pasteurization substantially reduces the number of living microorganisms, including probiotics. This could explain the unexpected results regarding the sauerkraut microbiota. Therefore, one could conclude that the used sauerkraut was a synbiotic in the sense that it contained pre- and postbiotics, and not probiotics.

## 5. Conclusions

The present study confirms the concept that sauerkraut supplementation could potentially be used in sports nutrition for gut microbiota optimization. The microbiota of organic, pasteurized sauerkraut is rich in anaerobic bacteria. The benefits of sauerkraut intake for active athletes are favorable changes in the gut microbiota composition and functionality, in the form of increased numbers of health-promoting bacteria and improved functionality. These could result in an enhanced immune response with increased lymphocytes, as well as a decreased metabolism of potentially pathogenic facultative anaerobes, resulting in decreased vitamin B12. But since sauerkraut supplementation poses a risk of indigestion, a seven-day-long adaptation period is required. In a follow-up study, these results have to be reproduced in a greater number of subjects and tested for sustainability by adding a wash-out period. In the future, sauerkraut brine could be investigated and supplementation provided for a longer time.

## Figures and Tables

**Table 1:** Nutritional data per 100 g of sauerkraut.

Calories	80 kJ/81 kcal
Protein	0.8 g
Carbohydrates	3.61 g
Sugar	0.2 g
Fat	0.1 g
Salt	1.99 g
Fiber	1.5 g

kJ—kilojoule; kcal—kilocalorie; g—gram.

**Table 2:** Description of the participants (N = 10).

(a) Personal Information						
Participant	Gender	Age (Years)	Sport	Years in Sport	Participant Classification Framework	
1	M	27	Karate	20	Tier 5	
2	M	37	Table tennis	31	Tier 5	
3	F	28	Bodybuilding	20	Tier 2	
4	M	38	Triathlon	30	Tier 3	
5	M	30	Kayaking/Kanu/ Rafting	23	Tier 5	
6	M	26	Triathlon	13	Tier 2	
7	M	27	Kayaking/Kanu/ Rafting	19	Tier 5	
8	M	23	Soccer	18	Tier 3	
9	M	27	Bodybuilding	12	Tier 4	
10	M	27	Kayaking/Kanu/ Rafting	21	Tier 5	
Average value, SD	29 ± 4.81		20.7 ± 6.18			
(b) Physical characteristics and body composition variables of participants						
Participant	Height (cm)	Body mass (kg)	FFM (kg)	SMM (kg)	BF (%)	FM (kg)
1	193.0	88.4	76.2	44.8	13.8	12.2
2	185.0	80.9	69.7	40.3	13.9	11.2
3	168.3	60.0	-	-	-	-
4	177.5	73.0	64.5	37.9	11.7	8.5
5	190.0	99.0	80.8	48.2	18.4	18.2
6	190.0	87.2	79.3	48.6	9.1	7.9
7	188.0	99.1	77.0	44.5	22.3	22.1
8	180.0	79.4	66.1	39.0	16.8	13.3
9	185.0	108.5	89.3	58.3	17.7	19.2
10	184.0	87.5	71.2	41.5	18.6	16.3
(c) Data on physical activity and sleep/						
			Before intervention	During intervention	Difference (*p*-value)	
Training frequency (per week)			6.22 ± 2.28	6.22 ± 2.63	1.000	
Training duration (minutes per day)			54.22 ± 36.94	61.61 ± 38.13	0.104	
Sleep time (hours)			7.74 ± 0.77	8.06 ± 0.82	0.073	
(d) Daily dietary intake						
			Before intervention (average, SD)	During intervention (average, SD)	Difference (*p*-value)	
Energy intake (kcal)			2741.59 ± 660.90	2747.72 ± 1017.84	0.983	
Protein intake (g)			158.29 ± 42.08	160.49 ± 55.55	0.868	
Protein intake (g/kg)			1.84 ± 0.29	1.84 ± 0.47	0.995	
Carbohydrate intake (g)			293.39 ± 100.09	267.14 ± 76.32	0.288	
Carbohydrate intake (g/kg)			3.48 ± 1.22	3.14 ± 0.95	0.208	
Fat intake (g)			94.63 ± 19.10	97.25 ± 38.06	0.813	
Fat intake (% energy intake)			31.39 ± 3.41	31.66 ± 2.14	0.854	
Fiber intake (g)			21.91 ± 5.89	25.09 ± 5.02	0.111	
Fiber intake (g/1000 kcal)			8.20 ± 2.28	9.98 ± 3.10	0.030 *	
(e) ADI Results						
Score (maximum points)			N	Before intervention (average, SD)	During intervention (average, SD)	Difference (*p*-value)
Special Nutrients subscale (35)			7	18.4 ± 2.2	17.4 ± 3.2	0.448
Core Nutrition subscale (80)			7	47.6 ± 12.9	46.9 ± 8.7	0.860
Dietary Habits (10)			7	6.6 ± 1.6	6.1 ± 1.5	0.289
Overall Score (125)			10	72.2 ± 12.5	71.4 ± 10.6	0.937
Overall Score (%)			10	57.7 ± 9.8	57.1 ± 8.6	0.863

M: male, F: female, SD: standard deviation. FFM, fat-free mass; SMM, skeletal muscle mass; BF, percentage of body fat; FM, fat mass. SD: standard deviation, * *p* < 0.05. N: number of participants who reported; SD: standard deviation.

**Table 3:** (a) Repeated measure correlation on gut microbiota functionality. (b) Alterations in modules of gut microbiota functionality.

(a)			
Pathway	*p*-Value	Correlation Coefficient	FDR
pyrimidine deoxyribonucleotides de novo biosynthesis I	0.000	−0.928	0.026
pyrimidine deoxyribonucleotide phosphorylation	0.000	−0.925	0.026
superpathway of guanosine nucleotides de novo biosynthesis I	0.001	−0.916	0.026
superpathway of pyrimidine ribonucleosides salvage	0.001	−0.911	0.026
superpathway of guanosine nucleotides de novo biosynthesis II	0.001	−0.909	0.026
pyrimidine deoxyribonucleotides de novo biosynthesis II	0.001	−0.898	0.026
superpathway of pyrimidine ribonucleotides de novo biosynthesis	0.001	−0.896	0.026
superpathway of purine nucleotides de novo biosynthesis I	0.001	−0.894	0.026
superpathway of purine nucleotides de novo biosynthesis II	0.001	−0.889	0.026
pyrimidine deoxyribonucleotides de novo biosynthesis III	0.001	−0.889	0.026
superpathway of pyrimidine deoxyribonucleotides de novo biosynthesis (*E. coli*)	0.002	−0.878	0.032
superpathway of pyrimidine deoxyribonucleoside salvage	0.002	−0.874	0.033
peptidoglycan maturation (meso-diaminopimelate containing)	0.004	0.852	0.052
superpathway of glycolysis and Entner-Doudoroff	0.005	−0.835	0.066
superpathway of thiamin diphosphate biosynthesis I	0.005	−0.834	0.066
glycogen degradation I (bacterial)	0.009	0.806	0.104
superpathway of L-alanine biosynthesis	0.010	0.796	0.116
superpathway of N-acetylneuraminate degradation	0.011	−0.791	0.118
galactose degradation I (Leloir pathway)	0.015	0.773	0.140
thiamin salvage II	0.015	0.772	0.140
sucrose degradation III (sucrose invertase)	0.016	0.767	0.142
superpathway of histidine, purine, and pyrimidine biosynthesis	0.016	−0.764	0.142
gluconeogenesis I	0.022	0.744	0.163
sucrose degradation IV (sucrose phosphorylase)	0.022	0.741	0.163
S-adenosyl-L-methionine cycle I	0.022	0.741	0.163
NAD salvage pathway I	0.023	0.740	0.163
CMP−3-deoxy-D-manno-octulosonate biosynthesis I	0.024	−0.737	0.163
superpathway of hexuronide and hexuronate degradation	0.024	0.735	0.163
TCA cycle VI (obligate autotrophs)	0.032	−0.709	0.208
superpathway of fucose and rhamnose degradation	0.033	0.708	0.208
L-glutamate and L-glutamine biosynthesis	0.035	0.702	0.214
superpathway of &beta;-D-glucuronide and D-glucuronate degradation	0.037	0.697	0.214
L-rhamnose degradation I	0.038	0.695	0.214
starch degradation V	0.038	0.694	0.214
superpathway of pyrimidine deoxyribonucleotides de novo biosynthesis	0.043	−0.683	0.232
(b)			
Module	*p*-value	correlation coefficient	FDR
Polysaccharide degradation	0.019	0.755	0.142
Sugar degradation	0.026	0.729	0.142
Indicators of inflammation	0.041	−0.687	0.142
Protein fermentation	0.043	0.681	0.142
Butyrate metabolism	0.047	−0.672	0.142

FDR: false-discovery-rate-corrected *p*-values.

**Table 4:** Laboratory parameters.

Parameter	Unit	Before Intervention (Mean, SD)	Day after Intervention (Mean, SD)	*p*-Value
Testosterone	(mg/mL)	19.14 ± 6.84	23.60 ± 10.04	0.363
Cortisol	(mg/mL)	500.8 ± 87.69	498.51 ± 144.44	0.974
Folic acid	(mg/mL)	25.67 ± 5.58	25.48 ± 5.49	0.882
Vitamin B12	(mg/mL)	394.1 ± 101.1	356.9 ± 108.1	0.012 *
Vitamin D	(mg/mL)	74.48 ± 12.82	80.2 ± 11.96	0.37
beta	(%)	114.84 ± 38.33	115.38 ± 45.36	0.974
S	(%)	145.19 ± 50.61	103.35 ± 46.2	0.054
IR	(%)	0.93 ± 0.46	0.94 ± 0.42	0.971
Insulin	(pmol/L)	55.51 ± 33.47	56.16 ± 30.09	0.846
TSH	(mIU/L)	2.94 ± 1.45	3.69 ± 2.13	0.146
T3	(pmol/L)	5.21 ± 1.11	5.11 ± 1.07	0.233
CRP	(mg/L)	0.65 ± 0.24	0.57 ± 0.11	0.466
LDL cholesterol	(mmol/L)	3.39 ± 1.33	3.08 ± 1.13	0.239
Urate	(μmol/L)	285.4 ± 38.7	290.6 ± 44.32	0.824
Lymphocytes	(%)	42.04 ± 3.764	45.55 ± 5.38	0.022 *
Neutrophils	(%)	47.39 ± 3.89	45.17 ± 6.99	0.221
Leukocytes	(109/L)	6.05 ± 1.31	6.02 ± 1.14	0.937
Erythrocytes	(1012/L)	4.96 ± 0.23	4.99 ± 0.18	0.681

SD: standard deviation; * *p* < 0.05.

**Table 5:** Bowel movement and adverse effects.

	Participants (N) Indicating BTS						Probability for BTS 3 and 4		Participants (N) Indicating Adverse Effects		
Day	1	2	3	4	5	6	HR, CI, *p*-Value	Bloating	Diarrhea	Pain	Constipation
1	0	4	3	2	0	1	50%, [18.7%, 81.3%], 1.000	1	1	1	0
2	0	3	2	2	3	0	40%, [12.2%, 73.8%], 0.527	1	0	0	0
3	0	2	0	5	3	0	50%, [18.8%, 81.3%], 1.000	2	0	0	0
4	0	2	2	4	2	0	60%, [26.2%, 87.8%], 0.527	1	0	0	0
5	0	1	2	4	2	1	60%, [26.2%, 87.8%], 0.527	3	1	1	0
6	0	2	1	4	1	2	50%, [18.7%, 81.3%], 1.000	3	2	1	0
7	0	2	2	2	4	0	40%, [12.2%, 73.8%], 0.527	2	0	0	0
8	0	0	2	8	0	0	100%, [69.2%, 100%], 0.002 *	1	0	0	0
9	0	1	1	8	0	0	100%, [55.5%, 99.7%], 0.011 *	1	0	0	0
10	0	0	2	8	0	0	100%, [69.2%, 100%], 0.002 *	1	0	0	0

N: 10, CI: 95% confidence interval, * *p* < 0.05.

## Data Availability

The original contributions presented in this study are included in the article. Further inquiries can be directed to the corresponding author.
